# The Promise of Inferring the Past Using the Ancestral Recombination Graph

**DOI:** 10.1093/gbe/evae005

**Published:** 2024-01-18

**Authors:** Débora Y C Brandt, Christian D Huber, Charleston W K Chiang, Diego Ortega-Del Vecchyo

**Affiliations:** Department of Genetics Evolution and Environment, University College London, London, UK; Department of Biology, Pennsylvania State University, University Park, PA, USA; Center for Genetic Epidemiology, Department of Population and Public Health Sciences, Keck School of Medicine, University of Southern California, Los Angeles, CA, USA; Department of Quantitative and Computational Biology, University of Southern California, Los Angeles, CA, USA; Laboratorio Internacional de Investigación sobre el Genoma Humano, Universidad Nacional Autónoma De México, Querétaro, Querétaro, Mexico

**Keywords:** ancestral recombination graph, demographic inference, natural selection

## Abstract

The ancestral recombination graph (ARG) is a structure that represents the history of coalescent and recombination events connecting a set of sequences (Hudson RR. In: Futuyma D, Antonovics J, editors. Gene genealogies and the coalescent process. In: Oxford Surveys in Evolutionary Biology; 1991. p. 1 to 44.). The full ARG can be represented as a set of genealogical trees at every locus in the genome, annotated with recombination events that change the topology of the trees between adjacent loci and the mutations that occurred along the branches of those trees (Griffiths RC, Marjoram P. An ancestral recombination graph. In: Donnelly P, Tavare S, editors. Progress in population genetics and human evolution. Springer; 1997. p. 257 to 270.). Valuable insights can be gained into past evolutionary processes, such as demographic events or the influence of natural selection, by studying the ARG. It is regarded as the “holy grail” of population genetics (Hubisz M, Siepel A. Inference of ancestral recombination graphs using ARGweaver. In: Dutheil JY, editors. Statistical population genomics. New York, NY: Springer US; 2020. p. 231–266.) since it encodes the processes that generate all patterns of allelic and haplotypic variation from which all commonly used summary statistics in population genetic research (e.g. heterozygosity and linkage disequilibrium) can be derived. Many previous evolutionary inferences relied on summary statistics extracted from the genotype matrix. Evolutionary inferences using the ARG represent a significant advancement as the ARG is a representation of the evolutionary history of a sample that shows the past history of recombination, coalescence, and mutation events across a particular sequence. This representation in theory contains as much information, if not more, than the combination of all independent summary statistics that could be derived from the genotype matrix. Consistent with this idea, some of the first ARG-based analyses have proven to be more powerful than summary statistic-based analyses (Speidel L, Forest M, Shi S, Myers SR. A method for genome-wide genealogy estimation for thousands of samples. Nat Genet. 2019:51(9):1321 to 1329.; Stern AJ, Wilton PR, Nielsen R. An approximate full-likelihood method for inferring selection and allele frequency trajectories from DNA sequence data. PLoS Genet. 2019:15(9):e1008384.; Hubisz MJ, Williams AL, Siepel A. Mapping gene flow between ancient hominins through demography-aware inference of the ancestral recombination graph. PLoS Genet. 2020:16(8):e1008895.; Fan C, Mancuso N, Chiang CWK. A genealogical estimate of genetic relationships. Am J Hum Genet. 2022:109(5):812–824.; Fan C, Cahoon JL, Dinh BL, Ortega-Del Vecchyo D, Huber C, Edge MD, Mancuso N, Chiang CWK. A likelihood-based framework for demographic inference from genealogical trees. bioRxiv. 2023.10.10.561787. 2023.; Hejase HA, Mo Z, Campagna L, Siepel A. A deep-learning approach for inference of selective sweeps from the ancestral recombination graph. Mol Biol Evol. 2022:39(1):msab332.; Link V, Schraiber JG, Fan C, Dinh B, Mancuso N, Chiang CWK, Edge MD. Tree-based QTL mapping with expected local genetic relatedness matrices. bioRxiv. 2023.04.07.536093. 2023.; Zhang BC, Biddanda A, Gunnarsson ÁF, Cooper F, Palamara PF. Biobank-scale inference of ancestral recombination graphs enables genealogical analysis of complex traits. Nat Genet. 2023:55(5):768–776.). As such, there has been significant interest in the field to investigate 2 main problems related to the ARG: (i) How can we estimate the ARG based on genomic data, and (ii) how can we extract information of past evolutionary processes from the ARG? In this perspective, we highlight 3 topics that pertain to these main issues: The development of computational innovations that enable the estimation of the ARG; remaining challenges in estimating the ARG; and methodological advances for deducing evolutionary forces and mechanisms using the ARG. This perspective serves to introduce the readers to the types of questions that can be explored using the ARG and to highlight some of the most pressing issues that must be addressed in order to make ARG-based inference an indispensable tool for evolutionary research.

**Significance**

The history of coalescence, mutation, and recombination events between a set of sequences is represented in a structure known as the ancestral recombination graph (ARG). The ARG is very informative of past evolutionary history and this property has generated a lot of interest in the development of methodologies that leverage the ARG. Here we discuss methodologies to infer the ARG, challenges remaining to estimate the ARG, and how we can use the ARG to infer past evolutionary processes.

## Methods to Estimate the ARG

**Fig. 1.**
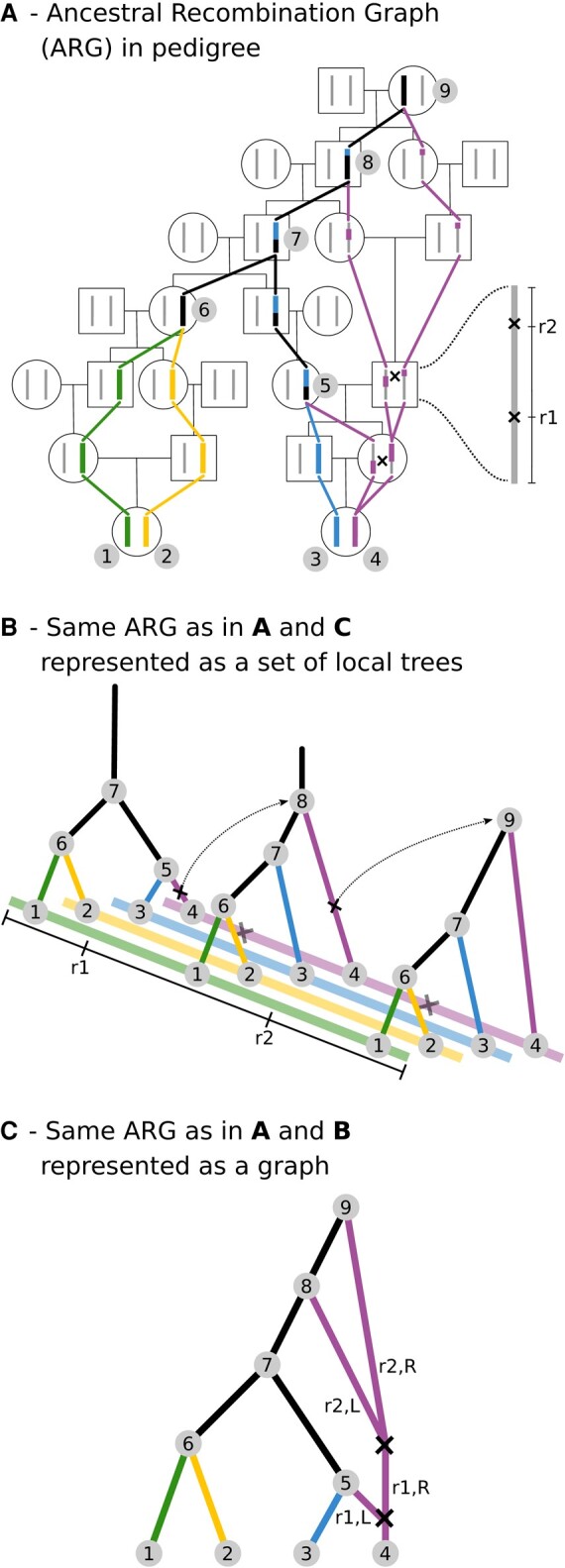
Schematic representations of the genealogy of a sample of 2 diploid individuals. Colors represent the 4 haplotypes sampled, and black lines indicate lineages or sequence tracts where at least 1 coalescence has occurred. Black crosses indicate recombination events. a) The genealogy or ARG embedded in a pedigree. We note that the pedigree representation shows a large amount of inbreeding (i.e. very small effective population size), unlikely to be found in outbreeding natural populations. The pedigree is used here solely for illustration purposes, to explicitly show the process of coalescence as lineages merging and recombination as lineages splitting, as we look backward in time. b) The same full ARG represented as a set of correlated local trees separated by a single recombination event. c) An equivalent representation of the full ARG as a graph that represents all genealogical relationships shown in a) and b), given that branches leading to recombination nodes are annotated with the corresponding sequence coordinates: left (L) or right (R) of the first (r1) or second (r2) recombination site. Figure modified from Brandt et al. (2022).

The estimation of the ancestral recombination graph (ARG) is a challenging problem since it requires 3 parts: the delimitation of sections of the genome that share the same history, showing the genealogical history in each section of the genome and highlighting genealogical changes between adjacent sections of the genome due to recombination (Fig. 1). We note that genealogical changes between adjacent sections of the genome occur more frequently between more ancient branches of the ARG because those branches tend to be longer. This property is due to the recombination events appearing on the analyzed sequences with a rate that is positively correlated with the product of the branch length multiplied by the recombination rate per base. When a recombination event takes place, it changes the coalescent patterns on branches between adjacent sections of the genome. As a corollary, the more recent nodes of an ARG tend to be shared among longer sections of the genome because those branches tend to be shorter. It must be noted that past demographic events affect these factors. As an example, a recent small population history produces more long sections of the genome sharing the same history and a higher amount of haplotype sharing (see Deng et al. 2021 for the theoretical distribution of the distances between changes in shared history). Developing methods that can estimate the ARG at a genome-wide scale “accurately” is a complex computational problem as the number of possible ARGs grows rapidly with increasing sample sizes. Additionally, there are different ways of coding an ARG that have been recently formalized and well explained on a recent paper (Wong et al. 2023). The accuracy of the inferred ARGs can be measured with respect to estimates of (i) local tree topologies, (ii) coalescent times and their uncertainties, and/or (iii) recombination breakpoints and transitions between adjacent trees, including the sharing of nodes across consecutive local trees, which translates to haplotype sharing in the data. Ideally, an ARG estimation method should provide benchmark tests showing how well they estimate these components of the ARG, but this has not been systematically adopted in the literature.

The development of methods to estimate the ARG has been ongoing since 1990 when the first approaches, based on parsimony, were introduced (Hein 1990). However, these methods had limitations in terms of accuracy, the number of chromosomes, and the length of the sequence that could be used to estimate the ARG. Since then, approximately 20 methodologies have been developed to estimate the ARG, with some of these methods extending the functionality of others (Minichiello and Durbin 2006; Rasmussen et al. 2014; Mirzaei and Wu 2017; Heine et al. 2018; Kelleher et al. 2019; Speidel et al. 2019; Hubisz et al. 2020; Ignatieva et al. 2021; Schaefer et al. 2021; Speidel et al. 2021; Mahmoudi et al. 2022; Wohns et al. 2022; Zhang et al. 2023). Currently, the most significant developments in ARG estimation have been made along 2 separate domains: increasing accuracy and improving scalability (number of samples and/or length of sequence analyzed). Thus far, most methods have faced a trade-off between accuracy and scalability that limited simultaneous advances in both areas. Future methods that can simultaneously improve both accuracy and scalability will be key to realize the promises of ARG-based inference. In this perspective, we will highlight 3 particularly noteworthy methods: ARGweaver (Rasmussen et al. 2014), Relate (Speidel et al. 2019), and tsinfer/tsdate (Kelleher et al. 2019; Wohns et al. 2022). We will examine the algorithmic foundations of these 3 methods and discuss significant advances proposed by other ARG estimation methods. We decided to highlight ARGweaver (Rasmussen et al. 2014), Relate (Speidel et al. 2019), and tsinfer/tsdate (Kelleher et al. 2019; Wohns et al. 2022) (i) because their performance to estimate ARGs has been assessed by independent peers not involved in the creation of the original methods (Brandt et al. 2022), (ii) because they have been widely used by the community, and (iii) due to the significant advances on scalability or accuracy of the estimated ARGs done by those methods. We note that methods that have been developed in the past couple of years hold promise to make large improvements on scalability or accuracy such as ARGinfer (Mahmoudi et al. 2022) and ARG-needle (Zhang et al. 2023). We hope to see more future work comparing the performance of these newer methods in contrast to previous methods.

ARGweaver (Rasmussen et al. 2014) greatly improved the accuracy of the estimated ARGs by implementing a new strategy called threading to navigate the ARG space in order to perform Markov chain Monte Carlo (MCMC) based estimation. This strategy is based on the sampling of an ARG of *N* chromosomes from an ARG of *N*-1 chromosomes. This approach has its roots in the conditional sampling distribution framework (Paul and Song 2010), which gives the probability of sampling a new sequence given a set of *N*-1 observed sequences. The authors showed that this sampling is computationally tractable using the assumptions of the sequentially Markovian coalescent (SMC) model (McVean and Cardin 2005). Briefly, the SMC model assumes that recombination breaks the genome into segments that share the same topology and that neighboring segments differ by a single change in the coalescence node of a particular branch. Crucially, the SMC model simplifies the “coalescent with recombination” model by assuming that any local genealogy only depends on the previous genealogy along the sequence (i.e. the Markovian assumption). ARGweaver's threading operation removes and reattaches a new branch to the topology of each segment in a manner consistent with the SMC model, thereby estimating the ARG that best explains patterns of genetic variation in the *N* sampled sequences. ARGweaver provides a very accurate reconstruction of the ARG as seen both in simulation and in comparison with other methods (Rasmussen et al. 2014; Brandt et al. 2022). In fact, the inaccuracies in ARGweaver results are due to 3 characteristics under a demographic model with a constant population size: approximations of the SMC model, the discretization of time, and MCMC convergence. Those 3 characteristics should be the main focus for methods aiming to achieve higher accuracy than ARGweaver under a demographic model with a constant population size. The estimation of ARGs under alternative demographic models to a constant population size model is another topic that should be inspected by ARG inference methods. Another advantage of ARGweaver is that it provides samples from a posterior distribution of ARGs instead of a single point estimate of it, providing the user with a measure of uncertainty around the estimated ARGs and lending itself well for downstream evolutionary methods that can take this uncertainty into account. Relate and tsinfer/tsdate, on the other hand, provide posterior samples of coalescent times but under a fixed estimate of the ARG topology.

Despite its accuracy, ARGweaver is limited to reconstructing the ARG of dozens of chromosomes (Rasmussen et al. 2014). Two recent methods enabled ARG estimation on thousands of samples: Relate (Speidel et al. 2019) and tsinfer (Kelleher et al. 2019). Relate employs a modified version of the Li–Stephens model (Li and Stephens 2003) to calculate the genetic distance between all haplotypes at each segment of the genome that share the same topology. Those distances are then used to reconstruct the genealogies across all segments in the genome. On the other hand, tsinfer first reconstructs “ancestral sequences” of a set of sampled chromosomes and then infers the relationship between the sequences using a variation of the Li–Stephens model (Li and Stephens 2003). Both the Relate and tsinfer/tsdate methods greatly increase the scalability of ARG estimation methods, with Relate (Speidel et al. 2019) being capable of handling up to ∼10,000 genomes and tsinfer/tsdate (Kelleher et al. 2019) being capable of handling >100,000 genomes. However, both tsinfer/tsdate and Relate are less accurate than ARGweaver in estimating coalescence times (Brandt et al. 2022). Their reduced accuracy is likely due to the approximations of the Li–Stephens model and heuristics used by these methods, which do not approximate the coalescence with recombination as well as the SMC model. However, it is crucial to note that the approximations used by tsinfer and Relate are expected to work better with larger sample sizes. Since ARGweaver is not scalable to the same order of magnitude of sample size, a direct comparison of accuracy at these larger sample sizes is not feasible.

ARGweaver, Relate, and tsinfer were the first methods developed based on defined models that simplify the coalescent with recombination (namely, SMC and Li and Stephens models). Nevertheless, there have been other innovative approaches developed to estimate the ARG apart from Relate, tsinfer, and ARGweaver (Table 1). These include, but are not limited to, a set of methods using heuristic approximations of the coalescent model with recombination to obtain plausible reconstructions of the ARG (Minichiello and Durbin 2006), improved parsimony-based ARG reconstruction methods (Ignatieva et al. 2021), fast heuristic methods to reconstruct local genealogies (Mirzaei and Wu 2017), a new approach to parallelize the computations inside a chromosome to estimate the ARG (Heine et al. 2018), a method that leverages the succinct tree sequence data structure (Kelleher et al. 2018) to speed up a Bayesian estimation of the ARG (Mahmoudi et al. 2022), approaches to estimate the ARG of a large number of individuals using genotype array data (Zhang et al. 2023), and extensions of ARG estimation methods to incorporate information from aDNA (Speidel et al. 2021) and to infer archaic gene flow events (Hubisz et al. 2020; Schaefer et al. 2021). These various approaches aim to improve general ARG estimation speed, scalability, or accuracy or tackle the inclusion of more specific data types, such as genotyping array data or ancient samples.

**Table 1:** Methods for estimation of ancestral recombination graphs

Method	Main challenge addressed	Key innovation	Limitations	Framework
Margarita (Minichiello and Durbin 2006)	Scalability (thousands of individuals, hundreds of SNPs)	More principled than previous haplotype clustering, faster than fully model-based methods	Maximizes tract lengths, probably underestimating recombinations	Heuristics based on CwR and SMC
ARGweaver (Rasmussen et al. 2014)	Accuracy	Navigating ARG space with threading algorithm. Provides estimates of uncertainty.	Inference done using a set of specified times, scalability (10s of whole genomes)	SMC or SMC”
RENT+ (Mirzaei and Wu 2017)	Scalability (faster than ARGweaver for the same number of samples)	Estimate trees per SNP and then merge compatible consecutive trees	Only provides point estimate	Heuristics
Arbores (Heine et al. 2018)	Speed (through parallelization of computations)	Parallelization with tree-bridging MCMC sampler. Provides estimates of uncertainty.	Scalability (tested on less than 10 sequences)	SMC
Relate (Speidel et al. 2019, 2021)	Scalability (up to 10^4^ genomes), inclusion of ancient samples	Two step estimation (topology, then coalescence times), has an aDNA extension. Provides estimates of coalescence times uncertainty.	Fixed topologies	L&S
tsinfer/tsdate (Kelleher et al. 2019; Wohns et al. 2022)	Scalability (10^5^ genomes), speed	Tree sequence encoding and 2 step estimation (topology and then coalescence times). Provides estimates of coalescence times uncertainty.	Fixed topologies, Inference done using a set of prespecified times	L&S
ARGweaver-D (Hubisz et al. 2020)	Assumption of constant population size and structure	Includes demography in model and provides estimates of uncertainty.	Inference done using a set of prespecified times, scalability	SMC or SMC’
SARGE (Schaefer et al. 2021)	Scalability (500 genomes)	Fast algorithm to find best branch movement to explain failure of the 4-gamete test	Only provides point estimate and minimizes recombination events	Parsimony
KwARG (Ignatieva et al. 2021)	Estimation with recurrent mutations	Parsimony (minimal recombination) heuristics allowing for variable amounts of recombination and/or recurrent mutation	Scalability (∼10s sequences, ∼1,000s bp)	Parsimony
ARGinfer (Mahmoudi et al. 2022)	Accuracy	Augmented tree sequence encoding and probabilistic estimation under the CwR. Provides estimates of uncertainty.	Scalability (∼10s sequences, ∼100s kb)	CwR
ARG-Needle (Zhang et al. 2023)	Accuracy (among the more scalable methods like Relate and tsinfer/tsdate)	ASMC clustering followed by sequence threading and ARG normalization for better calibration of posteriors	Accuracy (has not been compared to other highly accurate methods like ARGweaver or ARGinfer)	ASMC

We highlight the main challenge addressed by each method, their key innovation, limitations, and the model or framework on which each method is based on. Model names are abbreviated as follows: CwR (coalescent with recombination), SMC (sequentially Markovian coalescent), L&S (Li and Stephens algorithm; Li and Stephens 2003). ASMC is the Ascertained Sequentially Markovian Coalescent algorithm (Palamara et al. 2018).

## Challenges

Despite significant advances in the methodologies for reconstructing ARGs, there are still considerable trade-offs to be made to balance their accuracy and scalability. While some methods, such as ARGweaver (Rasmussen et al. 2014), are among the most accurate, they are not scalable for large data sets. On the other hand, methods like tsinfer (Kelleher et al. 2019) can handle a large number of samples but sacrifice accuracy. Future methods for ARG estimation need to be both accurate and scalable to overcome these limitations. Currently, these challenges limit the applicability of ARG estimation methods to infer evolutionary events, highlighting the need for further development in this area (Brandt et al. 2022).

ARG estimation is challenging because the space of possible ARGs is very large and thus hard to explore. Current methods that estimate the ARG use a simplified model, such as the SMC model, that allows a faster exploration of the ARG space while allowing the calculation of tractable likelihoods. In principle, it is possible that the development of mathematical models that make further simplifications of the ARG could allow for a faster navigation of the ARG space while permitting the calculation of tractable likelihoods and, therefore, allow for faster inferences of the ARG. The accuracy of these ARG inferences will depend on the impact of the simplifications of these models.

The evaluation of accuracy of ARG estimation methods per se is also challenging. As mentioned earlier, the accuracy of the estimated ARG can be quantified based on local tree topologies, coalescent times, or the sharing of nodes between consecutive trees. However, most often, a researcher's goal is to use ARG estimation methods to understand underlying evolutionary processes and not necessarily to directly estimate an ARG most similar to the true one. Therefore, the appropriate metric for accuracy may differ depending on the intended downstream application. For example, inferences about demographic history that depend on whole-genome patterns of coalescence rates might be better with methods that can rely on large sample sizes and whole genomes. On the other hand, inferences about selection parameters might gain more from methods that more precisely estimate the local trees at the selected loci. Therefore, the choice and accuracy of the ARG reconstruction method will ultimately be dictated by the intended evolutionary parameter one wishes to study.

Moreover, a better understanding of how different evolutionary processes and the misspecification of evolutionary parameters can impact the ARG estimation is still necessary. For instance, both background selection and positive selection can reduce the total branch length and alter the shape of genealogies (Rasmussen et al. 2014; Ortega-Del Vecchyo et al. 2022). Other evolutionary parameters that could be impacting ARG estimation, if misspecified, are mutation and recombination rates—both are heterogeneous across the genome and likely heterogeneous over time (Stapley et al. 2017; DeWitt et al. 2021), as well as the mispolarization of ancestral allelic states (Hernandez et al. 2007). As a result, the number of inferred genealogies in a given segment and the branch lengths over time within a genealogy could be biased. As an example, underspecified recombination rates would produce a larger number of bases with a shared genealogical history. On the other hand, an underspecified mutation rate would decrease the inferred coalescent rates. The impact of the mispolarization of ancestral states on the ARG reconstruction is a topic that needs further investigation.

Furthermore, it is worth noting that most ARG reconstruction methods have been developed for diploid, sexually reproducing organisms, and may not be directly applicable to organisms with different ploidy or reproductive mechanisms. For instance, different ARG reconstruction methods are needed for bacteria to take into account clonal reproduction and horizontal gene transfer (Vaughan et al. 2017) since it generates an asymmetry in the contributed DNA from parents to their offspring at recombination events, which is better approximated by the coalescent with gene conversion than the coalescent with recombination (Vaughan et al. 2017). Different ARG reconstruction methods will also be needed to analyze self-fertilizing organisms such as *Arabidopsis thaliana* or polyploid plants. In the case of polyploid plants, ARG reconstruction is complicated because haplotype phasing becomes more difficult as the number of chromosomes increases (Schrinner et al. 2020). Haplotype phasing errors due to statistical phasing can be a problem if the ARG reconstruction methods require prephased haplotypes (Kelleher et al. 2019; Speidel et al. 2019). Haplotype phasing could also bias ARG reconstruction if the method averages over all possible phasings including phasings that are incorrect (Rasmussen et al. 2014).

Finally, current ARG reconstruction methods tend to be based on 2 main assumptions often made in population genetics. The first assumption, which determines the shape of the genealogies, is that the sequences coalesce following the standard coalescent model. The second assumption, which determines the amount of variation we see on each site, is that there are only 2 possible alleles per site. Regarding the first assumption, the standard coalescent model may not be applicable in scenarios where multiple mergers are common, such as in many marine organisms with sweepstakes reproductive success (i.e. high variance in offspring number) (Hedgecock and Pudovkin 2011; Zhu et al. 2015). It remains to be explored how ARG inference using the standard coalescent or its approximations as a prior is affected when the true evolutionary model is more similar to a multiple merger coalescent (Tellier and Lemaire 2014). Previous results suggest that past population sizes can be underestimated if there are unaccounted multiple mergers in the data (Bhaskar et al. 2014). Regarding the second assumption, large sequencing studies on humans have identified a large number of recurrent mutations (Harpak et al. 2016; Lek et al. 2016) and segregating sites with more than 2 alleles (Lek et al. 2016). Some methods assume that there can be recurrent mutations per site, e.g. ARGweaver (Rasmussen et al. 2014), while others assume that there can be only 1 mutation per site, e.g. Relate (Speidel et al. 2019). In the future, it will be necessary to take into account multiallelic sites in ARG estimation methods that are currently discarded to infer the ARG.

## Downstream Evolutionary and Statistical Genetic Applications

The analysis of the inferred ARG provides a unique opportunity to gain a deeper understanding of past evolutionary processes. Many past methods treated the ARG as a latent variable to perform inferences to identify regions under positive selection (Huber et al. 2016) or to characterize evolutionary processes such as the past demographic history (Excoffier et al. 2021). Methods that use the information encoded on the ARG make use of the fact that all summary statistics of genetic variation in the data are, in the end, functions of the ARG. In particular, the coalescent rates over time encoded on an inferred ARG provide a rich source of information to analyze phenomena such as temporal changes of allele frequencies, the impact of natural selection, or past population structure.

One of the first evolutionary applications of the inferred ARG to understand the past was the inference of genetic adaptation. An early approach to do this was developed with ARGweaver, where an ARG-derived summary statistic was used to differentiate between 2 types of selection that were hard to distinguish with previous methods: background selection and selective sweeps (Rasmussen et al. 2014). CLUES uses another approach based on a parametric model of how an allele under recent positive selection affects the genealogy to infer the likelihood and strength of natural selection from the observed genealogy at a locus (Stern et al. 2019). A more recent machine learning method, SIA (Hejase et al. 2022), builds on the ideas of CLUES along with the selection statistics to detect signatures of positive selection developed in the software Relate (Speidel et al. 2019). Based on this foundation, SIA incorporates information from nearby (i.e. linked neutral) genealogies to more accurately infer the impact of natural selection (Hejase et al. 2021).

In addition, the inferred ARG is a valuable tool for analyzing past demographic events. The first example of an application to estimate the ARG and the past demographic history is the popular program PSMC that estimates the past population history based on a sample of 2 chromosomes from an individual (Li and Durbin 2011). Similarly, the software Relate and Colate can use the coalescent rates from samples of many individuals to infer past population sizes and population structure through time (Speidel et al. 2019, 2021). Additionally, ARGweaver-D is an extension of ARGweaver that can estimate the ARG under a prespecified demographic model to detect archaic introgressions (Hubisz et al. 2020). A composite likelihood method estimates population sizes and migration rates employing information of coalescent rates from subtrees of 3 individuals from larger genealogies inferred across the genome (Pope et al. 2023). Another recent method can also infer demographic parameters under complex models that include past population sizes, divergences, and admixtures by employing a graph-based structure to efficiently compute the exact marginal probability of coalescent trees with thousands of haplotypes (Fan et al. 2023). The inferred ARG has also been leveraged to analyze past population structure via the computation of an expected genetic relationship matrix defined as eGRM (Fan et al. 2022) as the ARG inherently leverages linkage information to depict the relationship between the sampled individuals. Incorporating geographic information can further enhance the analysis of the inferred ARG and help characterize population structure via the inference of dispersal rates (Battey et al. 2020; Osmond and Coop 2021) and the location of genetic ancestors of sampled individuals (Osmond and Coop 2021; Wohns et al. 2022).

Furthermore, the inferred ARG can enhance our understanding of the genotype–phenotype relationship for complex traits. For instance, researchers have studied the evolution of complex traits by combining the inferred ARG with genome-wide association studies data to analyze how directional selection has potentially shaped the evolution of phenotypic traits (Speidel et al. 2019; Stern et al. 2020) or to analyze the evolution of polygenic scores (Edge and Coop 2019). The inferred ARG and the GRM derived from it can also improve the robustness and power of association analysis to identify novel trait-associated loci, particularly in under-resourced populations or under complicated models of genetic architecture such as allelic heterogeneity (Link et al. 2023; Zhang et al. 2023).

Across these applications, 2 broad categories of analysis currently leveraging the inferred ARG emerge. One is based on computing the expectation of a statistic from the inferred ARG (Ralph et al. 2020) as in the case of the eGRM (Fan et al. 2022; Link et al. 2023; Zhang et al. 2023). Another category of analyses uses a model-based approach to estimate an evolutionary parameter of interest as in CLUES (Stern et al. 2019), PALM (Stern et al. 2020), or SIA (Hejase et al. 2021). Despite the substantial computational cost, explicit modeling is the more principled approach to test and estimate the parameters of an evolutionary model. As such, we would expect future development to focus on making model-based approaches more efficient and flexible. These approaches could open previously intractable applications and offer a powerful alternative to infer past evolutionary processes based on the genomic data and the ARG from many samples.

## Conclusion and Future Prospects

The development of methods capable of estimating the ARG opens the door to new analyses that interpret the joint patterns of coalescent and recombination events encoded in the ARG to understand our past. Such methods should lead to more accurate inferences of past evolutionary processes of interest that would be hard to pinpoint using only traditional statistics based on patterns of genetic variation. In particular, the ARG explicitly introduces the temporal dimension that is usually missing from raw genetic variation data. Therefore, more accurate estimation of the ARG will make possible a principled approach to, for example, infer temporal changes on the impact of natural selection or the changing pattern of population structure over time. We expect to see further developments on ARG-based analysis that will paint a more detailed picture of evolutionary processes acting on patterns of genetic and phenotypic variation.

## Supplementary Material

evae005_Supplementary_Data

## Data Availability

There are no new data associated with this article.

## Supplementary Material

Supplementary material containing chinese, portuguese and spanish translations of the abstract are available at *Genome Biology and Evolution* online.
