# Supplementary material for: The Promise of Inferring the Past Using the Ancestral Recombination Graph
Source: Genome Biol Evol. 2024 Jan 18;16(2):evae005. doi: 10.1093/gbe/evae005 (PMC10834162; doi:10.1093/gbe/evae005)
Supplement: evae005_Supplementary_Data [file evae005_supplementary_data.zip › GBE_abstract_chinese.docx]

**Traditional Chinese**

祖先重組圖（Ancestral Recombination Graph, ARG）是一種用來表示遺傳序列之間溯祖（coalescence）和重組（recombination）事件的歷史的結構（Hudson 1991）。一個完整的祖先重組圖可以表述為一系列於基因組上位點對應的家譜樹（genealogical trees），包括導致相鄰家譜樹之間的結構變化的重組事件，以及位於家譜樹支桿上的突變（ Griffiths & Marjoram 1997）。透過研究祖先重組圖，可以對演化過程得到許多有價值的認識，例如族群事件（demographic events）或自然選擇的發生與影響。祖先重組圖編碼了產生所有基因型和單倍型背後的過程，所有常用的群體遺傳學統計量（雜合率、連鎖不平衡等）都可以由它推導出來，因此它又被稱為群體遺傳學的「聖杯」（Hubisz & Siepel 2020）。歷史上，許多關於演化過程的推論都依賴從基因型矩陣中提取出的統計量。基於祖先重組圖的演化推測將會是一個重大的進步，因為它包含了重組、溯祖、突變等基因型中無法直接得到的數據，理論上其資訊量等同於甚至超過所有基於基因型的統計量的組合。正如預期，比起基於統計量的分析，最新的一些基於祖先重組圖的分析已經表現出一定的優勢（Stern et al. 2019; Speidel et al. 2019; Hubisz et al. 2020; Hejase et al. 2022; Fan et al. 2022, 2023; Link et al. 2023; Zhang et al. 2023）。因此，關於祖先重組圖的兩個問題的探討顯得尤為矚目：1）怎樣透過基因組資料來推測祖先重組圖；2）怎樣從祖先重組圖中獲得關於演化過程的資訊？在本文中，我們圍繞這些問題重點討論三個方面：推測祖先重組圖所需的計算方法上的革新；關於推測祖先重組圖仍然需要解決的一些挑戰；使用祖先重組圖推導出過程的動力和機制的方法論進展。本文向讀者介紹哪些類型的問題可以透過祖先重組圖來探討；也強調一些為使基於祖先重組圖的推論成為演化學研究不可或缺的工具亟待解決的問題。

**Simplified Chinese**

祖先重组图（Ancestral Recombination Graph, ARG）是一种用来表示遗传序列之间溯祖（coalescence）和重组（recombination）事件的历史的结构（Hudson 1991）。一个完整的祖先重组图可以表述为一系列於基因组上位点对应的家谱树（genealogical trees），包括导致相邻家谱树之间的结构变化的重组事件，以及位于家谱树支杆上的突变（Griffiths & Marjoram 1997）。通过研究祖先重组图，可以对演化过程得到许多很有价值的认识，例如族群事件（demographic events）或自然选择的发生与影响。祖先重组图编码了产生所有基因型和单倍型背后的过程，所有常用的群体遗传学统计量（杂合率、连锁不平衡等）都可以由它推导出来，因此它又被称为群体遗传学的“圣杯”（Hubisz & Siepel 2020）。历史上，许多关于演化过程的推断依赖于从基因型矩阵中提取出的统计量。基于祖先重组图的演化推测将会是一个重大的进步，因为它包含了重组、溯祖、突变等基因型中无法直接得到的数据，理论上其信息量等同于甚至超过所有基于基因型的统计量的组合。正如预期，比起基于统计量的分析，最新的一些基于祖先重组图的分析已经表现出一定的优势（Stern et al. 2019; Speidel et al. 2019; Hubisz et al. 2020; Hejase et al. 2022; Fan et al. 2022, 2023; Link et al. 2023; Zhang et al. 2023）。因此，关于祖先重组图的两个问题的探讨显得尤为瞩目：1）怎样通过基因组数据来推测祖先重组图；2）怎样从祖先重组图中获得关于演化过程的信息？在本文中，我们围绕这些问题重点讨论三个方面：推测祖先重组图所需的计算方法上的革新；关于推测祖先重组图仍然需要解决的一些挑战；使用祖先重组图推导演化过程的动力和机制的方法论进展。本文向读者介绍哪些类型的问题可以通过祖先重组图进行探索；也强调一些为使基于祖先重组图的推断成为演化学研究不可或缺的工具亟待解决的问题。
