# Supplementary material for: The Promise of Inferring the Past Using the Ancestral Recombination Graph
Source: Genome Biol Evol. 2024 Jan 18;16(2):evae005. doi: 10.1093/gbe/evae005 (PMC10834162; doi:10.1093/gbe/evae005)
Supplement: evae005_Supplementary_Data [file evae005_supplementary_data.zip › GBE_abstract_portuguese.docx]

The Portuguese translation of the abstract was done using Google Translate and corrected by Débora Y. C. Brandt, who is a native Portuguese speaker.

A tradução do resumo deste artigo para o português foi feita usando o Google Tradutor, seguida de correção manual por Débora Y. C. Brandt, que tem o português como língua materna.

O Grafo de Recombinação Ancestral (GRA, ou ARG na sigla em inglês) é uma estrutura que representa os eventos de coalescência e de recombinação passados que conectam um dado conjunto de sequências (Hudson 1991). O ARG completo pode ser representado como o conjunto das árvores genealógicas de cada locus do genoma, anotado com os eventos de recombinação que alteram a topologia das árvores entre loci adjacentes e as mutações que ocorreram ao longo dos ramos dessas árvores (Griffiths & Marjoram 1997). Informações valiosas sobre processos evolutivos passados, como eventos demográficos ou a influência da seleção natural, podem ser obtidas através do estudo do ARG. Ele é considerado o “Santo Graal” da genética populacional (Hubisz & Siepel 2020), uma vez que nele estão registrados os processos que geram todos os padrões de variação alélica e haplotípica a partir dos quais todas as estatísticas sumárias comumente usadas em genética de populações (por exemplo, heterozigose, desequilíbrio de ligação, etc.) podem ser derivadas. Até hoje, muitas inferências evolutivas basearam-se em estatísticas sumárias extraídas da matriz genotípica. As inferências evolutivas usando o ARG representam um avanço significativo, pois o ARG é uma representação da história evolutiva de uma amostra que inclui toda a história passada de eventos de recombinação, coalescência e mutação em uma dada sequência. Esta representação, em teoria, contém tanta informação quanto, se não mais, do que a combinação de todas as estatísticas sumárias independentes que poderiam ser derivadas da matriz genotípica. Consistente com esta ideia, algumas das primeiras análises baseadas no ARG revelaram-se mais poderosas do que análises baseadas em estatísticas sumárias (Stern et al. 2019; Speidel et al. 2019; Hubisz et al. 2020; Hejase et al. 2022; Fan e outros 2022, 2023; Link e outros 2023; Zhang e outros 2023). Deste modo, há grande interesse na área por duas importantes questões relacionadas ao ARG: 1) Como podemos estimar o ARG a partir de dados genômicos, e 2) Como podemos extrair do ARG informações sobre processos evolutivos passados? Nesta perspectiva destacamos três tópicos que dizem respeito a estas questões: O desenvolvimento de inovações computacionais que possibilitam estimar o ARG; desafios remanescentes na estimativa do ARG; e avanços metodológicos para deduzir forças e mecanismos evolutivos usando o ARG. Esta perspectiva tem o intuito de apresentar aos leitores os tipos de questões que podem ser exploradas usando o ARG e destacar algumas das questões mais urgentes que devem ser abordadas para tornar a inferência baseada no ARG uma ferramenta indispensável para a pesquisa evolutiva.
