# Supplementary material for: The Promise of Inferring the Past Using the Ancestral Recombination Graph
Source: Genome Biol Evol. 2024 Jan 18;16(2):evae005. doi: 10.1093/gbe/evae005 (PMC10834162; doi:10.1093/gbe/evae005)
Supplement: evae005_Supplementary_Data [file evae005_supplementary_data.zip › GBE_abstract_spanish.docx]

El grafo ancestral de recombinación (GAR) es una estructura que representa la historia de eventos coalescentes y de recombinación que conectan a un conjunto de secuencias (Hudson 1991). Anotado de forma completa, el GAR puede ser representado como un conjunto de árboles genealógicos en cada locus del genoma. En el GAR anotado de forma completa también se anotan los eventos de recombinación que cambian la topología de los árboles entre los loci adyacentes, además de las mutaciones que ocurren a lo largo de las ramas de dichos árboles (Griffiths y Marjoram 1997). Se pueden obtener descubrimientos valiosos de los procesos evolutivos del pasado, como los eventos demográficos en el pasado o la influencia de la selección natural, mediante el estudio del GAR. Al GAR se le conoce como el “santo grial” de la genética de poblaciones (Hubisz y Siepel 2020) ya que codifica los procesos que generan todos los procesos de variación alélica y haplotípica de los cuales se pueden derivar todos los “estadísticos de resumen” (e.g. heterocigosidad, desequilibrio de ligamiento, etc.) que se usan en la investigación en genética de poblaciones. Muchas inferencias evolutivas previas eran realizadas con un conjunto de estadísticos de resumen extraídos de la matriz de genotipos. Las inferencias evolutivas que usan el GAR representan un avance significativo porque el GAR es una representación de la historia evolutiva de una muestra que tiene la historia pasada de los eventos de recombinación, coalescencia y mutación a través de una secuencia particular. Esta representación en teoría contiene tanta información o más que la combinación de todos los estadísticos de resumen independientes que pueden ser obtenidos de la matriz de genotipos. De forma consistente con esta idea, algunos de los primeros análisis basados en el GAR han permitido análisis más poderosos que los análisis basados en estadísticos de resumen (Stern et al. 2019; Speidel et al. 2019; Hubisz et al. 2020; Hejase et al. 2022; Fan et al. 2022, 2023; Link et al. 2023; Zhang et al. 2023). Por ello, hay un interés significativo en el campo para investigar dos tópicos principales relacionados al GAR: 1) ¿Cómo podemos estimar el GAR basándonos en datos genómicos? y 2) ¿Cómo podemos extraer información de procesos evolutivos pasados a través del GAR? En esta perspectiva desarrollaremos tres temas que son relevantes para estos tópicos principales: El desarrollo de innovaciones computacionales que permitan la estimación del GAR; retos por resolver con respecto a la estimación del GAR; y avances metodológicos para deducir fuerzas y mecanismos evolutivos mediante el GAR. Esta perspectiva sirve para introducir a los lectores a los tipos de preguntas que pueden ser exploradas mediante el uso del GAR, además de subrayar algunos de los retos más importantes por resolver para hacer que las inferencias basadas en el GAR sean una herramienta indispensable para análisis evolutivos.
